# The Development and Validation of a Stability-Indicating UHPLC-DAD Method for Determination of Perindopril l-Arginine in Bulk Substance and Pharmaceutical Dosage Form

**DOI:** 10.1007/s10337-014-2724-7

**Published:** 2014-08-13

**Authors:** Magdalena Paczkowska, Przemysław Zalewski, Piotr Garbacki, Alicja Talaczyńska, Anna Krause, Judyta Cielecka-Piontek

**Affiliations:** 1 Department of Pharmaceutical Chemistry, Faculty of Pharmacy, Poznan University of Medical Sciences, Grunwaldzka 6, 60-780 Poznan, Poland; 2 PozLab sp.z.o.o, Poznan, Poland

**Keywords:** UHPLC-DAD, Perindopril, *cis–trans* isomers, Stability

## Abstract

A stability-indicating ultra-high-performance liquid chromatography (UHPLC) method with a diode array detector was developed and validated for the determination of *cis*/*trans* isomers of perindopril l-arginine in bulk substance and pharmaceutical dosage form. The separation was achieved on a Poroshell 120 Hilic (4.6 × 150 mm, 2.7 µm) column using a mobile phase composed of acetonitrile–0.1 % formic acid (20:80 *v/v*) at a flow rate of 1 mL min^−1^. The injection volume was 5.0 µL and the wavelength of detection was controlled at 230 nm. The selectivity of the UHPLC-DAD method was confirmed by determining perindopril l-arginine in the presence of degradation products formed during acid–base hydrolysis and oxidation as well as degradation in the solid state, at an increased relative air humidity and in dry air. The method’s linearity was investigated in the ranges 0.40–1.40 µg mL^−1^ for isomer I and 0.40–2.40 µg mL^−1^ for isomer II of perindopril l-arginine. The UHPLC-DAD method met the precision and accuracy criteria for the determination of the isomers of perindopril l-arginine. The limits of detection and quantitation were 0.1503 and 0.4555 µg mL^−1^ for isomer I and 0.0356 and 0.1078 µg mL^−1^ for isomer II, respectively.

## Introduction

**Fig. 1:**
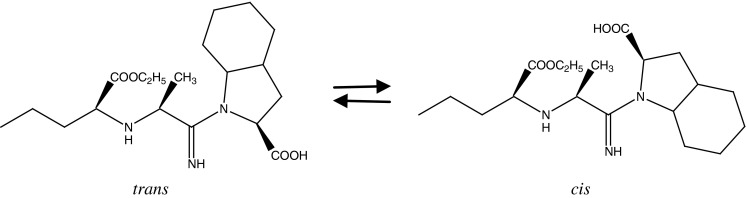
Chemical structure of *cis*/*trans* isomers of perindopril

Perindopril ((2*S*,3a*S*,7a*S*)-1-[(2*S*)-2-[[(2*S*)-1-ethoxy-1-oxopentan-2-yl]amino]propanoyl]-2,3,3a,4,5,6,7,7a-octahydroindole-2-carboxylic acid) is a long-acting angiotensin-converting enzyme (ACE) inhibitor which is effective in reducing blood pressure and improving outcomes in a number of cardiovascular diseases [1]. Perindopril is a prodrug ester which was observed to convert to diacid perindoprilat, an active metabolite, during in vivo studies [2]. Currently, two salts of perindopril, erbutamine (*tert*-butylamine) and l-arginine, are used therapeutically [3]. Both salts are chemically unstable and undergo degradation in dosage forms to other diacids and diketopiperazines [4]. Perindopril l-arginine is the first ACE inhibitor analog which contains only a stereoselective *S* enantiomers. Due to the presence of a double bond in the tertiary amide bond of the C-terminal proline containing a peptide bond, perindopril can exist in the form of *cis*/*trans* isomers (Fig. 1).

Only several HPLC-DAD methods have been reported for the determination of perindopril in the presence of its degradation products and synthesis-induced impurities [5, 6]. Since combinations of perindopril with drugs from other groups such as calcium channel blockers or diuretics are used in hypertension therapies, HPLC-DAD methods for the determination of perindopril in the presence of other drugs in pharmaceutical preparations and in body fluids have been reported as well [7, 8]. Chromatographic techniques have also been applied to investigate separation between the *cis* and *trans* isomers of perindopril. Stability studies of perindopril supported by a theoretical approach showed that the type of salt and the compound charge have an impact on its degradation, proving perindopril l-arginine to be more stable than erbutamine salt [9, 10]. No procedures based on UHPLC solutions for the determination of perindopril have been reported. However, the application of UHPLC method in perindopril determination, with reduction of analysis time with the possibility of a reliable separation of its isomers, meets the criteria of modern analytical techniques.

Therefore, the aim of our study was to develop a UHPLC-DAD method allowing separation of the *cis* and *trans* isomers in bulk substances and pharmaceutical dosage forms (Prestarium^®^), and to investigate their degradation under the influence of stress factors.

## Experimental

### Chemicals, Reagents, and Solutions

Perindopril l-arginine (purity >98 %) in bulk substance was applied by India PVT LTD., while Prestarium^®^, containing racemic mixture of *cis*/*trans* isomers (10 mg coated tablets), was a commercial preparation manufactured by Servier. Hydrochloric acid, sodium hydroxide solution, hydrogen peroxide and all other chemicals were obtained from P.O.Ch. (Gliwice, Poland). Acetonitrile of an HPLC grade was supplied by Merck KGaA (Darmstadt, Germany) and formic acid (100 %) by P.O.Ch. (Gliwice, Poland). Ultrapure water (18 MΩ cm at 25 °C) was prepared using an Exil SA 67120 Millipore purification system (Molsheim, France).

### Chromatographic Equipment

The LC system (Dionex Thermoline Fisher Scientific, Schwerte, Germany) was equipped with a high pressure pump (UltiMate 3000), an autosampler (UltiMate 3000) and a DAD detector (UltiMate 3000). For data processing and acquisition, Chromeleon software version 7.0 from Dionex Thermoline Fisher Scientific (US) was used. As the stationary phase, a Poroshell 120 Hilic column, 2.7 µm particle size, 4.6 × 150 mm (containing ultra-high purity silica, 99.995 % SiO_2_) (Agilent Technology, Santa Clara, US) was used. The mobile phase consisted of acetonitrile–0.1 % formic acid (20:80 *v/v*). The flow rate of the mobile phase was 1.0 mL min^−1^. The wavelength of the UV detector was set at 230 nm. The injection volume was 5.0 µL. The column temperature was 25 °C. Chromatographic separation was performed with a pressure of 420 bars. PH of mobile phase was measured by pH-meter (Mettler Toledo SevenCompact pH/Ion S220, Warsaw, Poland).

### Method Validation

The method was validated according to the International Conference on Harmonization Guidelines [11]. It comprised selectivity, linearity, accuracy, precision, limits of detection (LOD) and quantitation (LOQ). All measurements were performed in triplicates.

#### Sample Solutions

Sample solution of perindopril l-arginine was prepared by dissolving 10.0 mg of perindopril l-arginine in 25.0 mL of ultrapure water. Prestarium^®^, commercially available dosage form of perindopril l-arginine, was pounded and weighed mass of the appropriate 10.0 mg of perindopril l-arginine and dissolved in 25.0 mL of ultrapure water.

#### Selectivity

The selectivity of determination of perindopril l-arginine was examined for non-degraded and degraded samples. The selectivity of perindopril l-arginine determination was established in the presence of excipients (lactose monohydrate, magnesium stearate). While the selectivity of perindopril l-arginine determination in degraded samples was examined in the presence of degradation products formed during acidic–basic hydrolysis (in hydrochloric acid (1 M) at 353 K and in solution of sodium hydroxide (1 M) at 353 K), oxidation (10 % H_2_O_2_ at 353 K) and thermolysis (at an increased relative air humidity 76.4 % RH at 353 K and in dry air 0 % RH at 373 K).

#### Precision

Precision of the assay was determined in relation to repeatability (intra-day) and intermediate precision (inter-day) at three levels of concentration: 80 % (1.6 µg mL^−1^), 100 % (2.0 µg mL^−1^) and 120 % (2.4 µg mL^−1^). Every sample was injected six times.

#### Accuracy

The accuracy of the method was determined by recovering perindopril l-arginine from placebo. As placebo substance was chosen talc. The accuracy was established at three levels 80, 100 and 120 % of label claim of the substance for determination of perindopril l-arginine in bulk substance and in pharmaceutical dosage form.

#### The Limit of Detection (LOD) and Quantification (LOQ)

LOD and LOQ parameters were determined from the regression equations of perindopril l-arginine. LOD = 3.3 *S*_*y*_/*a*, LOQ = 10 *S*_*y*_/*a*; where *S*_*y*_ is a standard error and *a* is the slope of the corresponding calibration curve.

#### Robustness

The impact of the percentage of acetonitrile in the mobile phase, pH of mobile phase, the flow rate and the column temperature on the peak area and shape of perindopril l-arginine were determined.

#### Standard Solution

Standard stock solution of perindopril l-arginine was prepared by dissolving 10.0 mg of perindopril l-arginine in 25.0 mL of ultrapure water. Standard stock solution was kept during period of studies.

## Results and Discussion

**Fig. 2:**
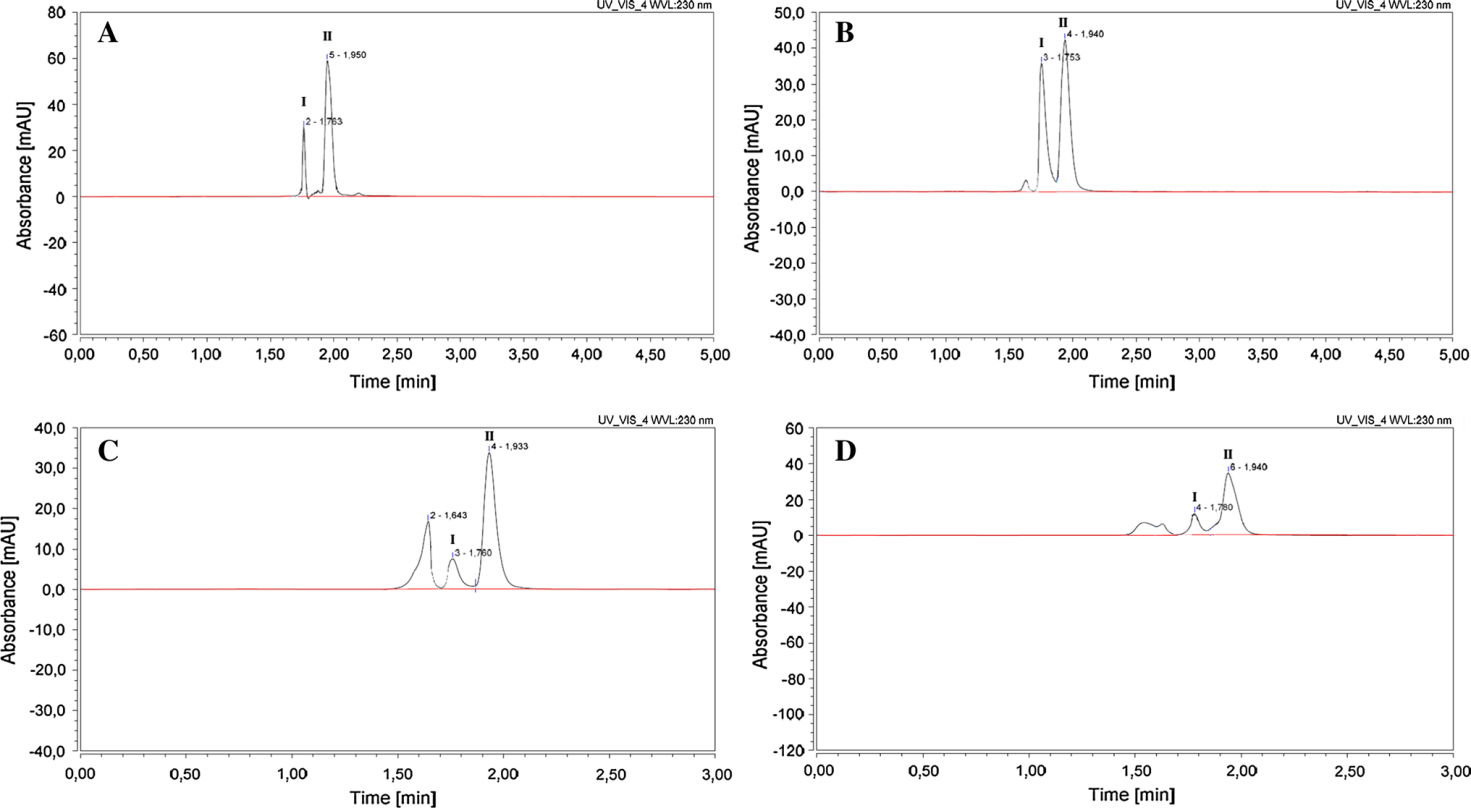
The chromatogram of isomer I (*t*_R_ = 1.763 min) and isomer II (*t*_R_ = 1.950 min) of perindopril l-arginine: in bulk substance (**a**); in pharmaceutical dosage form Prestarium^®^ (**b**); in degraded samples in HCl (1 M, *T* = 353 K, content of initial concentration 39 %) (**c**); in degraded samples in NaOH (1 M, *T* = 353 K, content of initial concentration 33 %) (**d**)

The UHPLC procedure was optimized with a view to developing a method for stability-indicating assay of perindopril l-arginine and separation of its *cis* and *trans* isomers in bulk substance and pharmaceutical dosage form. Under the optimized chromatographic conditions, two peaks for non-degraded samples of perindopril l-arginine were recorded. As shown by the chromatograms, isomers I and II of perindopril l-arginine were eluted at retention times *t*_R_ = 1.76 and *t*_R_ = 1.95, respectively (Fig. 2a). On the chromatograms of the degraded samples of perindopril l-arginine a decrease was observed in the peaks of the isomers and the appearance of peaks related to the degradation products was registered. The spectrophotometric purities of peaks of main substance in the presence of its degradation products were confirmed. Perindopril l-arginine was subjected to acidic, basic, thermal, photolytic, and oxidative stress conditions according to ICH regulations. Different rates of degradation were observed for the isomers depending on the stress factors applied. The peak tailing factors for the isomers of perindopril l-arginine were between 1.37 and 1.64. The chromatograms of non-degraded and degraded samples of perindopril l-arginine are shown in Fig. 2a–d. In a previous investigation, Bouabdallah et al. [5] confirmed the effect of organic solvent, pH, temperature, β-cyclodextrins and flow rate on the possibility of separating the *cis*/*trans* isomers of perindopril l-arginine when the chromatographic procedure was based on a column with 5 μm particle size. The isomers of perindopril l-arginine were eluted at 2.14 and 3.88 min. However, the isomers of perindopril l-arginine were better separated and in a shorter time when the UHPLC-DAD method described in the present work was applied.

The method was validated for selectivity, linearity, inter- and intra-day accuracy and precision, robustness, system suitability, LOD, LOQ and recovery for the assay of perindopril l-arginine in the pharmaceutical preparation Prestarium^®^. All validation parameters were established for the *cis*/*trans* isomers of perindopril l-arginine. The calibration plots were linear in concentration of isomer I in the range 0.40–1.40 µg mL^−1^ (*n* = 11, *r* = 0.9914) and isomer II 0.40–2.40 µg mL^−1^ (*n* = 11, *r* = 0.9998). They were described by the equations *y* = *ax*: *y* = (0.2143 ± 0.0394)*x* for isomer I and *y* = (1.7652 ± 0.0266)*x* for isomer II. The *b* values, calculated from the equation *y* = *ax* + *b,* were insignificant because of being lower than the critical value *t*_*b*_ = *b*/*S*_*b*_ (Table 1). The values of RSD for the determination of the isomers during the evaluation of intra-day precision ranged from 0.89 to 1.91 % for isomer I and from 1.06 to 1.98 % for isomer II. The ranges of inter-day determination were 1.04–1.71 and 1.20–1.80 % for isomers I and II, respectively. The accuracy of determination, established at 80, 100 and 120 % of the label claim, was in the ranges 95.56–96.27 % for isomer I and 97.10–97.89 % for isomer II. The LOD and LOQ for isomer I of perindopril l-arginine were 0.1503 and 0.4555 µg mL^−1^ while those for isomer II 0.0356 and 0.1078 µg mL^−1^, respectively.

**Table 1:** Validation parameters of linearity of isomer I and isomer II of perindopril l-arginine

	Isomer I	Isomer II
Retention time (min)	1.76	1.95
Range of linearity (µg mL^−1^)	0.40–1.40	0.40–2.40
Regression equation (*y*)		
Slope (a ± *S*_a_)	0.21 ± 0.04	1.77 ± 0.03
Intercept (b ± *S*_b_)	0.02 ± 0.04	0.01 ± 0.04
LOD (µg mL^−1^)	0.1503	0.0356
LOQ (µg mL^−1^)	0.4555	0.1078
Correlation coefficient (*r*)	0.9914	0.9998

*S*_*a*_ standard deviation of slope, *S*_*b*_ standard deviation of intercept, *t,* calculated values of Student’s *t* test, *t*_*α,f*_ = 2.1709 critical values of Student’s test for degrees of freedom *f* = 11 and significance level *α* = 0.05

The best separation of the *cis*/*trans* isomers of perindopril l-arginine was obtained for the above-mentioned chromatographic parameters. The robustness of the UHPLC-DAD procedure was evaluated after changing the mobile phase composition (acetonitrile volume, 20 ± 5 *v/v*), mobile phase pH (formic acid concentration, 3–0.05 %), flow rate (1.0 ± 0.1 mL min^−1^), absorption wavelength (353 ± 3 nm) and column temperature (25 ± 10 °C). No statistically significant differences were established for the parameters studied in the investigation of robustness as their deviation did not exceed the permissible range of ±2 %. While higher changes in these parameters resulted in the lack of separation of the isomers and the appearance of tailing peaks. System suitability was examined in regard to the relative standard deviation for peak areas and peak times, peak resolution, peak asymmetry and the number of theoretical plates. All parameters are collected in Table 2.

**Table 2:** System suitability tests of the UHPLC-DAD

Parameter	Isomer I	Isomer II
Relative standard deviation of peak area %	1.84	1.50
Relative standard deviation of retention time (min) %	0.02	0.02
Resolution	1.68	1.47
Asymmetry (tailing factor)	1.83	1.51
Number of theoretical plates	34,693	28,716

Resolutions were calculated between two adjacent peaks in no-degraded and degraded samples

## Conclusion

Since the UHPLC-DAD method presented in this communication is a simple, accurate, selective and time-saving tool for the determination of the *cis*/*trans* isomers of perindopril l-arginine, which also allows good separation, it can be used for routine testing and stability analysis of that compound. In addition, the method was validated for compliance with ICH guidelines and proved to meet the acceptance criteria. It may, therefore, be recommended for quality evaluation of perindopril l-arginine in the commercial product Prestarium^®^, including determination of its *cis*/*trans* isomers.
